# Arthroscopic Fixation of Comminuted Glenoid Fractures Using Cannulated Screws and Suture Anchors

**DOI:** 10.1097/MD.0000000000001923

**Published:** 2015-12-11

**Authors:** Feng Qu, Bangtuo Yuan, Wei Qi, Chunbao Li, Xuezhen Shen, Qi Guo, Gang Zhao, Jiangtao Wang, Hongliang Li, Xi Lu, Yujie Liu

**Affiliations:** From the Department of Orthopedics, Beijing Tongren Hospital, Capital Medical University (FQ); and Department of Orthopedics, Chinese PLA General Hospital, Beijing, China (FQ, BY, WQ, CL, XS, QG, GZ, JW, HL, XL, YL).

## Abstract

We investigate the feasibility of arthroscopic fixation of comminuted glenoid fractures using cannulated screws and suture anchors.

We retrospectively review 11 cases of closed comminuted glenoid fractures treated at our institution from August 2010 to May 2013. The 11 patients, 8 males and 3 females, had a mean age of 41 years (range: 27–55 years). The mechanisms of injury were traffic accidents in 9 cases and falls from height in 2 cases. The mean time from injury to surgery was 12 days (range: 3–28 days). All glenoid fractures were confirmed on x-ray and computed tomography. The major fracture fragments were fixed with cannulated screws and the small fragments were fixed with suture anchors.

All surgical wounds healed with primary closure and no complications including infection and neurovascular damage were observed. All 11 patients were followed up for a mean of 21 months (range: 14–29 months). Bone union was achieved in all patients with a mean time of 10 months. At the last follow-up, range of motion of the shoulder joint was significantly improved (*P* < 0.05). Both ASES scores (41.4 ± 24.9, 87.3 ± 13.8) and Rowe scores (28.2 ± 18.6, 93.2 ± 11.2) were significantly increased after the surgery (*P* < 0.01), indicating significantly improved function and stability of the shoulder joint.

Arthroscopic fixation using cannulated screws and suture anchors is feasible for the treatment of comminuted glenoid fractures. This method is minimally invasive and provides good functional recovery with a lower risk of complications.

## INTRODUCTION

The scapula is a component of the superior shoulder suspensory complex (SSSC) and bridges the upper limb to the axial skeleton through the clavicle. The glenoid cavity is a shallow pyriform cavity and articulates with the head of the humerus, forming the glenohumeral joint.^1^ Glenoid fracture is relatively rare, accounting for about 0.1% of whole body fractures and 10% of scapula fractures.^2^ Glenoid fractures are usually caused by high-energy impacts and are often associated with multiple injuries.^3^ These associated injuries may lead to treatment difficulties and missed diagnosis of the glenoid fracture.^4,5^ In addition, the bone fragments of glenoid fractures tend to displace due to the traumatic mechanism and muscular traction.^6^ Displaced glenoid fractures may result in unstable glenohumeral joints or luxation if not treated promptly and properly.^7–10^

The complex anatomy around the scapula poses a great challenge for open reduction and internal fixation (ORIF) of glenoid fractures. Anterior to the scapula is the brachial plexus as well as several large vessels; laterally are the circumflex scapular vessels, circumflex nerve, and posterior circumflex humeral vessels; and inferior to the acromion are the suprascapular nerve and suprascapular vessels. Therefore, ORIF treatment of glenoid fractures is associated with high risks of iatrogenic damage and poor surgical outcomes including decreased range of motion, pain, and an unstable shoulder joint.^11,12^ Shoulder arthroscopy is minimally invasive and has been used to manage a variety of shoulder disorders. We report our experience of using arthroscopic fixation of comminuted glenoid fractures using cannulated screws and suture anchors in a series of 11 cases.

## MATERIALS AND METHODS

### Patients

**FIGURE 1:**
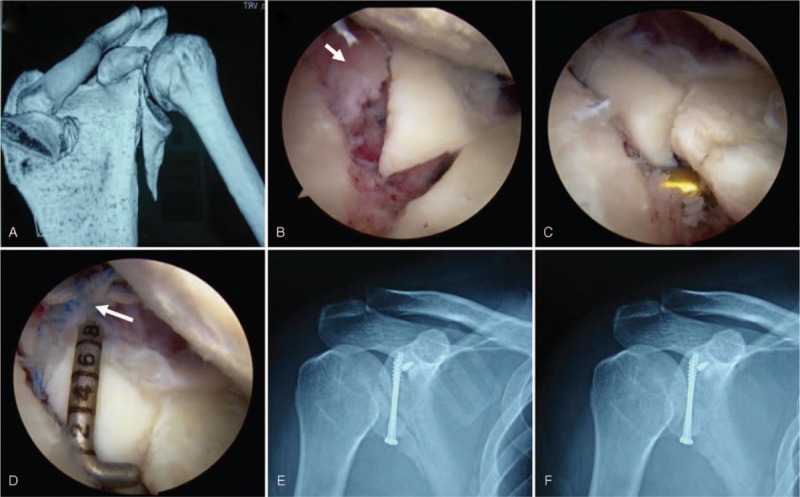
A, A 37-year-old man left suffered glenoid fracture after a traffic accident. A CT-3D image shows a displaced bone fragment. B, An arthroscopic image showing a small, displaced bone fragment near the fracture line (short arrow). C, Arthroscopic reduction of the bone fragments using a probe. D, The small bone fragment was fastened and fixed using the suture (long arrow). At postoperative 1 day (E) and 12 weeks (F), bone union was achieved. The X-ray films show the position of the cannulated screws and the suture anchor.

Eleven patients with closed comminuted glenoid fractures were treated at the Chinese General Hospital of the PLA from August 2010 to May 2013. These patients, 8 men and 3 women, had a mean age of 41 years (range: 27–55 years). The mechanisms of injury were traffic accidents in 9 cases and falls from height in 2 cases. The mean time from injury to surgery was 12 days (range: 3–28 days). In 5 cases the fractures were on the left side and were on the right side in the remaining 6 cases. Before surgery, range of motion of the shoulder was measured. The shoulder joints were also evaluated using the ASES and Rowe scales.^13^ All glenoid fractures were confirmed on x-ray and 3D reconstructed computed tomography (CT-3D) (Fig. 1A). All cases were operated on by the same team.

This study was approved by the Institutional Review Board of the Chinese PLA General Hospital. Each of the 11 patients provided written informed consent. All activities associated with this study were performed in accordance with the ethical standards of the institutional and/or national research committee and with the 1964 Helsinki declaration and its later amendments or comparable ethical standards.

### Surgical Procedure

Patients received general anesthesia and were positioned laterally. The injured upper limb was abducted 45 degrees and a 4-kg weight was used for traction. The shoulder joint was inflated and a portal was made at the “soft point,” 1.5 cm inferior and 1.5 cm medial to the posterolateral margin of the acromion. The arthroscope was inserted through the portal and an anterior working passage was established using the Outside-In technique with the arthroscope. The glenoid fracture was evaluated using a probe to determine the degree of displacement and cartilage damage (Figure 1B). Scar tissue was resected and a probe was inserted along the margin of the fracture between the major bone fragment and the scapula. The bone fragment was manipulated, using the probe, toward the opposite direction of displacement and reduced (Figure 1C). The manipulation point was selected at the superior margin of the bone fragment, far away from any nerves or vessels. Manipulation of the bone fragment was performed gently to avoid damage to any surrounding structure. After confirmation of reduction, 2 to 3 parallel K-wires were inserted perpendicular to the fracture line. A 2.5-mm-diameter drill was used to fashion holes in the bone and 2 to 3 cannulated screws were inserted across the fracture line. For the fixation of the small displaced bone fragments, a suture anchor was inserted into the base of the bone fragment and then pulled out and fastened around the fragment to achieved fixation (Figure 1D). Tears of the glenoid labrum and the rotator cuff were sutured and damaged cartilage was cleaned. The articular cavity was rinsed several times and reduction and fixation were confirmed with radiography (Figure 1E).

### Postoperative Management and Evaluation

The operated shoulder was protected using a shoulder abduction brace (RS-B-01, Beijing Oriental Resun Prosthetics & Orthotics Technology Development Co., Ltd, Beijing, China) for 4 weeks. Passive movement was initiated 3 days after the surgery and strength exercises were initiated 2 months postoperatively. Patients were followed up by clinic visits and the shoulder joints were evaluated using the ASES and Rowe scales. Range of motion was also assessed including abduction, flexion, and external rotation.

### Statistical Analysis

All data were expressed as mean ± standard deviation and compared using a paired *t* test. Statistical analyses were performed using SPSS 13.0 software (SPSS Inc, Chicago, IL). Statistical significance was considered when *P* < 0.05.

## RESULTS

All the surgical wounds healed with primary closure and no postoperative complications including infection or neurovascular injury were observed. All patients were followed up for a mean of 21 months (range: 14–29 months). X-ray examinations of all patients showed bone union (Figure 1F), with a mean time from surgery to union of 10 weeks (range: 8–13 weeks). At the last follow-up, range of motion of the shoulder was significantly improved in comparison to preoperative findings (Table 1). Both ASES and Rowe scores were significantly improved postoperatively, suggesting significantly improved function and stability of the shoulder joint (Table 2).

**TABLE 1:**
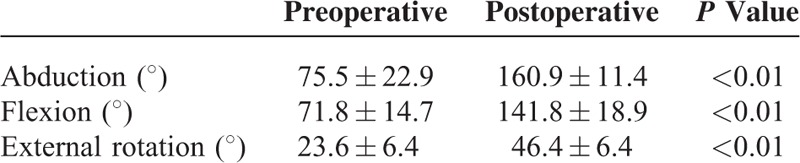
Preoperative and Postoperative Range of Motion of the Shoulder Joint

**TABLE 2:**
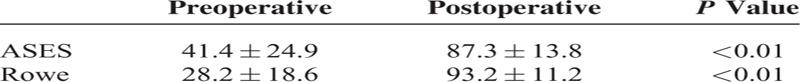
Preoperative and Postoperative Evaluation of the Shoulder Joint

## DISCUSSION

Shoulder arthroscopy is minimally invasive and can help avoid injury to nerves and vessels during surgical exposure. Additionally, arthroscopy does not require stripping of the periosteum and is therefore beneficial for promoting bone union. The view of the surgical field provided by arthroscopy is magnified and can show precise details. Magnification of the surgical field is also useful in reducing small bone fragments. Shoulder arthroscopy only requires a small portal in the skin and preserves the integrity of the joint capsule, which can help avoid irritation caused by adhesion in the capsule.^14^ In addition, the muscles surrounding the shoulder joint are minimally affected by arthroscopy. In our series of cases, the fracture was fixed using 2 screws, which provided anti-rotation capabilities. Therefore, patients could begin functional exercises in the early stages after surgery while minimizing the risks of postoperative disuse and complications.^15,16^ Arthroscopy also provides the capability for simultaneous repair of injuries to the tendons, rotator cuff, and glenoid labrum, which can maximize recovery of joint function.^17^

In our experience, fixation of small fractured bone fragments is difficult with cannulated screws. Suture anchors are relatively small in volume, provide an adequate holding force on the bone fragments, and do not affect the insertion of cannulated screws. In this study, small bone fragments were fastened and fixed with the sutures. This technique was reliable and did not cause breakage of the bone fragments. Anatomical reduction was achieved in all cases using the suture anchors. For the reduction and fixation of the comminuted intra-articular fractures, full evaluation of the fracture and displacement is necessary. CT-3D reconstruction is an effective method for preoperative evaluation of the fractures and surgical planning.^18^ All patients herein were evaluated using CT-3D images to understand the distribution and displacement of the fracture fragments.

The ASES scale is a commonly used tool for evaluation of shoulder joint function and has been verified in terms of reliability, validity, sensitivity, and responsiveness.^19^ The Rowe scale is often used to evaluate long-term results of glenoid labrum surgeries. The scores of stability account for 50% in the Rowe scale, which makes the Rowe scale suitable for evaluation of shoulder joint stability, especially in cases of glenoid fractures which can greatly affect joint stability. Therefore, restoration of joint stability is critical for successful surgical management. The postoperative ASES and Rowe scores in the present study were significantly increased postoperatively, indicating a significant improvement in function and stability of the shoulder joint.

## CONCLUSION

Our study demonstrates that arthroscopic fixation of comminuted glenoid fractures using cannulated screws and suture anchors is both safe and effective. This method might result in reduced complications and improved functional outcomes when compared with open reduction.
